# Barriers to implementation of guidelines for the diagnosis and management of undescended testis

**DOI:** 10.12688/f1000research.15532.1

**Published:** 2019-03-25

**Authors:** Shawn C. Smith, Hiep T. Nguyen

**Affiliations:** 1 Banner Children’s Specialists, Pediatric Urology, Cardon Children's Medical Center, 1432 S. Dobson Road, Suite #501, Mesa, AZ, 85202, USA

**Keywords:** American Urological Association, cryptorchidism, undescended testicles, guidelines

## Abstract

Cryptorchidism or undescended testis (UDT) is one of the most common genital disorders identified at birth. The gold standard for treatment in the US is to surgically bring the UDT into the scrotal sac. In 2014, the American Urologic Association (AUA) presented a guideline for evaluation and treatment of cryptorchidism. We reviewed some of the most recent domestic and international studies examining the results of implementing the AUA and similar guidelines for the diagnosis and management of UDT. In addition, we reviewed some of the more common barriers to the implementation of the AUA guidelines and offered recommendations on how to increase the rate of early detection of UDT, thereby increasing the rate of surgical correction at the appropriate age.

Cryptorchidism or undescended testis (UDT) is one of the most common genital disorders identified at birth. The gold standard for treatment in the US is to surgically bring the UDT into the scrotal sac. The main rationale for treatment is to affect the increased risks of infertility, testicular malignancy, torsion/trauma, and inguinal hernia associated with UDT. In 2014, the American Urologic Association (AUA) guidelines for evaluation and treatment of cryptorchidism were published ^1^. One of the main goals was to outline for the non-surgeon provider how to correctly identify bilateral and unilateral undescended testicles, at what age and under what circumstances to refer a boy with suspected or identified undescended testicles, and at what age persistent UDT should be surgically brought to the scrotum. In brief, the current AUA guidelines recommend that (1) at initial evaluation a full gestational history of all boys suspected of cryptorchidism be obtained; (2) the testicles be palpated at each recommended well-child visit for appropriate quality and position; (3) all infants who are found to have cryptorchidism at birth and who do not have spontaneous descent by age 6 months (corrected for gestational age) be referred to a surgeon for appropriate evaluation; (4) all boys with a possible new diagnosis of acquired cryptorchidism after 6 months of corrected gestational age be referred for possible surgical correction; (5) any phenotypic male newborns with bilateral non-palpable testes be referred for evaluation of possible disorder of sexual development (DSD); (6) ultrasound and other imaging studies (which are rarely sensitive diagnostic tools) not be ordered prior to referral to a surgical specialist; (7) severe proximal hypospadias and cryptorchidism alert the provider to assess for DSD; (8) a boy who is found to have bilateral non-palpable testes and who does not have congenital adrenal hyperplasia, Müllerian inhibiting substance, or other additional hormones be tested to evaluate for anorchia; and (9) at least annual physical exams be used to assess for secondary ascent in boys found to have retractile testicles.

Similar recommendations were offered by the Canadian Urological Association ^2^, the British Association of Paediatric Surgeons/British Association of Paediatric Urology Surgeons/Royal College of Surgeons ^3^, the Nordic Consensus Group ^4^, and the European Association of Urology/European Society for Paediatric Urology ^5^. The recommended age for orchidopexy was reduced to below 1 year on the basis of findings of germ cell loss in the UDT at 1 to 2 years of age ^4^ and findings that orchidopexy performed at 9 months compared with 3 years had a more significant beneficial effect on the growth of the previously undescended testes ^6^. Based on extensive review of the current literature ^1^, the use of intramuscular human chorionic gonadotropin (hCG) or intranasal gonadotropin-releasing hormone (GnRH) appears to be unreliable in inducing the descent of the UDT. However, the use of hormone therapy as an adjuvant with orchidopexy may help to improve the fertility of boys with UDT ^7, 8^.

Despite wide consensus by both pediatric urologists and pediatric surgeons nationally and internationally, there continues to be significant variation in the age when boys are referred for and undergo treatment for UDT. In a 2017 study from New Zealand, the authors evaluated boys with UDT seen at their institution between the time periods of 1996–1998 and 2014–2016 ^9^. The authors observed that there was a decrease in the median age at referral from 23 months in the 1996–1998 group to 5.3 months in the 2014–2016 group. There was also a decrease in median age of surgery from 38.8 months in the 1996–1998 group to 12.6 months in the 2014–2016 group. This decrease in the median age of referral and surgical correction was in line with the newest AUA guidelines. However, the majority of the other studies did not find a similar positive result. In a recently published German study of 5,547 boys with cryptorchidism seen at 16 hospitals nationwide between 2003 and 2016, the authors found that between 2003 and 2008 only 4% of all boys with UDT had surgical correction before the age of 1 year ^10^. This percentage changed only slightly after the German guideline update in 2009, increasing to 5% between 2010 and 2012 and then up to 8% between 2013 and 2016. Similarly, in a study from China, the median age of orchidopexy decreased from 3 years in 2010 to 2 years in 2015 ^11^. However, the target of recommended age of orchidopexy (prior to 1 year of age) was not achieved in any of the years that were studied. Correspondingly, in a 2017 study conducted in collaboration between West Virginia University and Johns Hopkins University, the authors evaluated 131 cases of UDT at an urban center and 100 at a rural center in the US ^12^. The average ages of referral were 48.3 months at the urban center and 59.6 months at the rural center; the average ages of surgical intervention were 53.8 and 65.2 months, respectively. Surgical correction at less than 18 months of age occurred in only 40% of the rural patients and 29% of the urban patients. Similarly, another study from the US indicated that only 18% of the patients with UDT underwent orchidopexy before the age of 2 years and 43% before the age of 3 years for the time period between 1999 and 2008 ^13^.

The aforementioned studies elucidate that, for some, lack of awareness and, for others, lack of understanding of the guidelines are critical reasons for the delay in the referral and management of UDT. In the German study, the authors surveyed physicians and medical students in regard to their knowledge about the guidelines ^10^. One third of the respondents did not know the guideline recommendations, and 61% felt insufficiently informed. In the Chinese study, the authors observed that in their survey of 305 primary health-care practitioners, only 64% would perform normal pediatric urology examination and only 26% would refer the patient to pediatric surgery prior to 1 year of age ^11^. In the US study, the delays in referral and treatment were similar in the urban and rural populations, suggesting that this issue is pervasive in both studied populations ^12^. Common reasons for the delayed referral from primary care providers include the following: not understanding the time frame to refer a patient with UDT to attain the benefit of surgical treatment; not performing follow-up genital exams during subsequent care visits; the misperception that it takes time (that is, more than 1 year) for the testicles to descend; the misinterpretation of the guidelines in believing that one has to wait until age 5 or later to refer; the misunderstanding that hormone therapy is first-line treatment instead of surgical intervention; and the misperception that scrotal ultrasound is a valid method for the detection of cryptorchidism that must be routinely performed prior to referral.

Education of the referring providers appears to help to decrease the median age in which patients with UDT are referred. In the Chinese study, the authors provided lectures and handouts to the primary health-care practitioners with regard to UDT guideline recommendations. Tracking of the age of orchidopexy revealed a statistically significant downward trend after this intervention was instituted ^11^. Similarly, a 2016 study carried out in England implemented an educational survey to 144 general practitioners, which provided them with current recommendations for the optimal time of referral and treatment for boys with UDT ^14^. The authors observed that the average age of referral improved significantly after educational intervention, decreasing from 2.8 years in 2010 to 1.25 years in 2013.

It is important to recognize that another reason for the late referral of boys with UDT is the development of acquired UDT such as from ascending testis and secondary cryptorchidism. Acquired UDT may account for up to 50% of all performed orchidopexies ^15^. In a 2013 study, van der Plas *et al*. documented that of the 660 patients who underwent orchidopexy after 2 years, 66% had at least two documented visits of the testis being in the scrotal position ^16^. Similarly, in a 2008 study, Guven and Kogan observed that 46% of boys who underwent orchidopexy after the age of 4 years had a previously documented retractile or scrotal position of the testis ^17^. As a consequence, the AUA guidelines and others have recommended yearly genital exam in boys in order to properly identify those patients with secondary UDT.

In a review of our office referral system, it was not difficult to find barriers to following the AUA and other guidelines’ recommendations. Some of these barriers are the following: a lack of access to pediatric surgeons/urologists and care-specific guidelines; the primary care provider’s misunderstanding of the physiology of undescended versus ascending testicles; lack of awareness that a thorough genital exam is essential at birth and as the child ages; the misconception that once the testicles are found in the scrotum they will always remain there; and the lack of parental follow-up evaluation or being lost to follow-up because of circumstances such as changing primary care physicians, family relocation, or insurance changes. Not only is it important that the primary care or referring providers be aware of the guidelines for the diagnosis and management of UDT, but the parents should also be educated about UDT. Consequently, it is vital that this information be provided in multiple formats so as to find as great a reach as possible. Furthermore, repetitive presentation of the guideline recommendations to both the providers and the parents will help to improve the understanding and ultimately the timely diagnosis of UDT.

As specialists taking care of boys with UDT, we have not been as effective in reaching primary care providers and the parents of the patients with the educational message compared with that of other diseases such as hypertension, heart disease, and cancer. This is due in part to the nature of the disease in that the number of patients affected with UDT is much smaller than those affected with the previously mentioned conditions. In addition, the decision for treatment of UDT is not made by the patient but by the parents or caregivers who may be an additional cause for the delay or even lack of treatment. Given these barriers, it is even more important for us specialists to make a greater effort, on a local as well as a national level, to educate the widest group of primary care providers and the parents of boys with UDT.

In conclusion, the age of referral and treatment of UDT remains higher than that recommended by the AUA and similar guidelines despite their international publication and awareness by specialists. The AUA and similar guidelines provide a clear and concise foundation for all providers on the prompt diagnosis and treatment of cryptorchidism. However, the level of knowledge about this condition by the primary care or referring providers and the parents remains in need of improvement. This is due in part to common misconceptions and misunderstandings about cryptorchidism and the failure to recognize secondary UDT. Increasing awareness through education in the form of lecture presentations, handouts, and web-based surveys can help to bridge this gap in knowledge. Only by addressing these issues can we improve the prompt diagnosis and treatment and improve the outcomes for boys with UDT.
